# Children With PANS May Manifest POTS

**DOI:** 10.3389/fneur.2022.819636

**Published:** 2022-04-26

**Authors:** Avis Chan, Jaynelle Gao, Madison Houston, Theresa Willett, Bahare Farhadian, Melissa Silverman, Paula Tran, Safwan Jaradeh, Margo Thienemann, Jennifer Frankovich

**Affiliations:** ^1^Division of Allergy, Immunology and Rheumatology, Department of Pediatrics, Stanford University School of Medicine, Palo Alto, CA, United States; ^2^Stanford PANS/Immune Behavioral Health Clinic and PANS Research Program at Lucile Packard Children's Hospital, Palo Alto, CA, United States; ^3^Department of Human Biology, Stanford University School of Humanities and Sciences, Stanford, CA, United States; ^4^Division of Child and Adolescent Psychiatry and Child Development, Department of Psychiatry and Behavioral Sciences, Stanford University School of Medicine, Palo Alto, CA, United States; ^5^Autonomic Disorders Program, Department of Neurology and Neurological Sciences, Stanford University School of Medicine, Palo Alto, CA, United States

**Keywords:** abrupt-onset obsessive compulsive disorder, autonomic dysfunction, autoimmunity, pediatric acute-onset neuropsychiatric syndrome (PANS), POTS

## Abstract

**Objectives:**

Pediatric acute-onset neuropsychiatric syndrome (PANS) is characterized by an abrupt-onset of severe psychiatric symptoms including OCD, anxiety, cognitive difficulties, and sleep issues which is thought to be a post-infection brain inflammatory disorder. We observed postural orthostatic tachycardia syndrome (POTS) which resolved with immunomodulation in a patient with Pediatric acute-onset neuropsychiatric syndrome (PANS). Here, we aim to present a case of POTS and to examine the prevalence of (POTS) in our PANS cohort, and compare the clinical characteristics of patients with and without POTS.

**Study Design:**

We conducted this cohort study of patients meeting PANS criteria who had at least three clinic visits during the study period. We included data from prospectively collected questionnaires and medical record review. We present a case followed by statistical comparisons within our cohort and a Kaplan-Meier analysis to determine the time-dependent risk of a POTS diagnosis.

**Results:**

Our study included 204 patients: mean age of PANS onset was 8.6 years, male sex (60%), non-Hispanic White (78%). Evidence of POTS was observed in 19/204 patients (9%) with 5/19 having persistent POTS defined as persistent abnormal orthostatic vitals, persistent POTS symptoms, and/or continued need for pharmacotherapy for POTS symptoms for at least 6 months). In this PANS cohort, patients with POTS were more likely to have comorbid joint hypermobility (63 vs 37%, *p* = 0.04), chronic fatigue (42 vs 18%, *p* = 0.03), and a family history of chronic fatigue, POTS, palpitations and syncope. An unadjusted logistic regression model showed that a PANS flare (abrupt neuropsychiatric deterioration) was significantly associated with an exacerbation of POTS symptoms (OR 3.3, 95% CI 1.4–7.6, *p* < 0.01).

**Conclusions:**

Our study describes a high prevalence of POTS in patients with PANS (compared to the general population) and supports an association between POTS presentation and PANS flare within our cohort.

## Introduction

Pediatric acute-onset neuropsychiatric syndrome (PANS) is a clinical syndrome characterized by the abrupt onset of obsessive-compulsive symptoms and other neuropsychiatric symptoms and is associated with neuroinflammation and thought to be post-infectious (1–12). The onset of PANS is dramatic, with patients commonly presenting overnight with obsessive compulsive symptoms or severely restricted food intake and at least two other similarly severe, abrupt-onset symptoms, including anxiety, emotional lability and/or depression, aggression, behavioral/developmental regression, cognitive impairment, sensory or motor abnormalities, or somatic handwriting changes and urinary symptoms. (13–15). Imaging studies point to pathology in the basal ganglia, including microstructural changes, microglial activation, and swelling during acute flares of PANS symptoms (6, 7, 12). PANS typically begins in early childhood and affects more boys than girls (16–22).

Studies have shown the association between some psychiatric disorders (including obsessive compulsive disorder [OCD] and tic disorders) and cardiovascular and autonomic dysfunction. (23–25). In children and adolescents, autonomic dysfunction may manifest as postural orthostatic tachycardia syndrome (POTS), (26, 27) which is characterized by orthostatic intolerance symptoms, including lightheadedness, palpitations, and an exaggerated heart rate increase occurring with postural change from lying to standing. POTS can be a debilitating condition causing physical, emotional, and social distress, and decreased quality of life (28). Identification of POTS is important, as pharmacologic, psychological (cognitive behavior therapy) and behavioral (yoga) interventions may improve symptoms (29, 30).

POTS affects approximately 1% of the general population, (31) often beginning in adolescence and predominantly affecting White females of childbearing age (32). An immunologic stressor is hypothesized to trigger the onset of POTS in some individuals (33). To our knowledge, no studies have examined the prevalence of POTS in patients with PANS, though both illnesses can present in childhood and adolescence, and have similar co-morbidities. In this paper, we describe the presenting symptoms and response to treatment in a patient with co-occurring POTS and PANS. We then examine the prevalence of POTS and factors associated with POTS in a cohort of patients with PANS. Lastly, we explore the association of POTS diagnosis with PANS flares (abrupt neuropsychiatric deteriorations). We hypothesized that POTS was more common in patients with PANS than the general population.

## Methods

### Study Design, Setting, and Participants

The Stanford Institutional Review Board approved this retrospective cohort study as part of a prospective study (IRB#26922). Assent was obtained from patients aged 7–17 years as well as written informed consent from parents of all minor patients.

This study was performed at the Stanford Immune Behavioral (IBH)/PANS Clinic between September 3, 2012 and June 30, 2019. Patients who declined research (*N* = 9), did not meet PANS criteria (*N* = 102), or had fewer than three clinic visits were excluded from this study (*N* = 39). The last exclusion criteria ensured adequate time for history exploration, physical examination, and detection of any autonomic abnormalities. The final study cohort included 204 patients.

### Chart Review and Patient Questionnaire

We reviewed medical records including those available within Stanford's electronic medical record (EMR) system, EPIC, and those from outside institutions. We collected data on patients' demographics; mental health/psychiatric history; orthostatic vital signs; POTS symptoms; comorbidities including joint hypermobility, headache, nausea and vomiting, non-specific abdominal pain, depression, anxiety, sleep disturbance, chronic fatigue, and cognitive impairment; medication history; and family history of known risk factors for POTS including fatigue, POTS, palpitations and syncope (26).

Prior to each clinic visit, parents and patients complete a questionnaire including psychometric rating scales and questions about the patient's interim medical and psychiatric symptoms and treatments (including all the data in the first paragraph). The psychometric scales include the Children's Yale-Brown Obsessive Compulsive Scale (CY-BOCS), a measure of OCD severity; (34) the Modified Overt Aggression Scale (MOAS), a measure of aggression; (35) and the Caregiver Burden Inventory (CBI), a measure of caregiver burden that has been validated in PANS patients (36, 37). At each visit, clinicians assessed the disease state and the global functioning of the child in the preceding week using the Children's Global Assessment Scale (CGAS) (38).

### Definitions of Measures

For this study, we used the Singer et al. (39) diagnostic criteria for POTS: the presence of orthostatic intolerance symptoms (lightheadedness or palpitations), occurring frequently when assuming the upright position, and an exaggerated heart rate increase associated with postural change from lying to standing. Orthostatic heart rate increase was considered exaggerated if the following criteria were met: (1) an increase over supine of ≥ 40 beats/min after 5–10 min of quiet standing or upright tilt, or (2) a sustained heart rate increase of ≥ 130 beats/min (for age ≤ 13 years) or ≥ 120 beats/min (for age > 13 years) present after adequate hydration (39). Persistent POTS was defined by persistent abnormal orthostatic vitals at least 6 months apart, along with persistent POTS symptoms or continuation of medications for their POTS symptoms. POTS was considered resolved when the patient had normal orthostatic vitals and no POTS symptoms.

### Other Factors for Investigation

To better understand the risk factors for POTS in our patients, we examined several laboratory and biometric markers, including body weight change in the preceding 3 months, urine specific gravity, and serum sodium and potassium levels at the time of orthostatic vital test. We chose these laboratory markers because increased urine specific gravity, hypernatremia and hyperkalemia may indicate hypovolemia and dehydration, both of which contribute to POTS symptoms. We recorded response to treatments, including immunomodulatory interventions (intravenous immunoglobulin [IVIG], methylprednisolone, prednisone use of >2 months duration, rituximab, methotrexate, and mycophenolate mofetil) and POTS treatments (salt tablets, fludrocortisone, pyridostigmine, midodrine, or beta blockers), for the period between the diagnosis and resolution of POTS.

### Statistical Analyses

For categorical variables, we made statistical comparisons using Chi-square tests, or Fisher's exact test when appropriate. For continuous variables, we conducted two-sample *t*-tests for normally distributed data and Wilcoxon rank-sum tests for skewed data. We also performed a Kaplan-Meier analysis to examine the time-dependent risk of POTS diagnosis. We used the Statistical Analysis Systems software program (SAS® University Edition, USA) for statistical analysis. All statistical tests were considered to be statistically significant if the two-sided *p* < 0.05.

## Case Presentation

A 14-year-old non-Hispanic White male was referred to our clinic for evaluation and treatment of a relapsing-remitting PANS illness followed by 4 years of persistent severe PANS symptoms, including severe OCD, tics, rage, and suicidality. In addition, he had multiple autoimmune diseases, including celiac disease, Hashimoto's thyroiditis with anti-thyroglobulin antibodies, and thrombocytopenia with anti-platelet antibodies. His family history was significant for Raynaud's disease in his father, celiac disease in maternal second degree family members, and OCD and bipolar disorder in paternal second degree family members. With previous neuropsychiatric deteriorations at a separate institution, he had been treated with high-dose IVIG, plasmapheresis, intravenous and oral corticosteroids, antibiotics, and multiple psychotropic medications.

His initial deterioration was at 10 years of age when he presented with abrupt-onset of OCD (contamination fears), eating restriction, anxiety, tics, rage, and cognitive difficulties coinciding with moderately high streptococcal titers in both the patient (anti-DNase B 328 [normal <170 U/mL), ASO 460.4 [normal < 200 U/mL]) and his brother. Prior to this deterioration, there were no mental health concerns. He was treated with monthly IVIG. Psychiatric symptoms gradually improved over 1.5 years, but following his 15th round of monthly IVIG he had a severe relapse, after which he was given two rounds of plasmapheresis. In the subsequent 2 years, he experienced three relapses characterized by a dramatic worsening of OCD, food restrictions, anxiety, rage, cognitive impairment, suicidal ideation, and POTS symptoms (described below).

At his initial presentation to our IBH/PANS clinic, the patient had chronically impairing obsessive compulsive symptoms (CYBOCS 32), suicidality, restricted food intake, anxiety, lightheadedness on standing (he preferred to be supine at all times), severe sleep disturbances characterized by multiple night awakenings, fatigue and he was not able to participate in school or extracurricular activities other than video games. Medications at the time of presentation to our clinic were: levothyroxine (50 mcg once daily) an melatonin (5 mg at night)—all other medications had been discontinued due to recent acetaminophen overdose (suicide attempt) and secondary liver dysfunction. His body mass index (BMI) was 17.5 kg/m^2^ (26th percentile). His orthostatic vitals were as follows: pulse 60 beats/min lying and 153 beats/min after quiet standing for 10 min; blood pressure 104/70 mmHg lying and 106/67 mmHg after quiet standing for 10 min. Heart rate was accelerated by 93/min on postural change, suggestive of POTS.

He was subsequently referred to a neuro-autonomic specialist for a formal tilt table test, which revealed a heart rate increase of 60 beats/min immediately after tilt, and then remained 45–58 beats above baseline, with a maximum heart rate of 136 beats/min, for 8 min of tilt. An increase in vasomotor pooling and lightheadedness were noted after the tilt. Delayed blood pressure response on Valsalva maneuver and decrease in his sweat responses over the feet suggested autonomic neuropathy.

Polysomnography (PSG) indicated central sleep apnea (apnea hypopnea index 21.5), delayed onset sleep and leg movements that did not meet periodic leg movement syndrome criteria.

Laboratory evaluation at clinic presentation (prior to immunomodulation) revealed lymphopenia (absolute lymphocyte count 0.9 K/μL [normal 1.5–6.5 K/μL]), thrombocytopenia (absolute platelet count 109 K/μL [normal 150–400 K/μL]), but normal hemoglobin and hematocrit. He had low complement: C3 (83 mg/dL [normal 86–184 mg/dL]) and C4 (12.8 mg/dL [normal 20–59 mg/dL]); but high C4a (7,211 ng/mL [normal < 2,830 ng/mL]). He had high autoantibodies to platelets, histone, and thyroglobulin, but the rest of the autoantibody work-up was negative including antinuclear antibodies and antiphospholipid antibodies. In sum, the patient met three out of four needed criteria for a diagnosis of systemic lupus erythematosus at clinic presentation: (1) neuropsychiatric symptoms; (2) lymphopenia (in the absence of recent immunomodulation), and (3) low C3/C4 complements.

In view of his multiple autoimmune diseases (PANS, thyroiditis, celiac disease) and markers of active autoimmunity (lymphopenia, thrombocytopenia, anti-platelet antibodies, anti-histone antibodies, anti-thyroid antibodies, and complement consumption/activation), we initiated immunomodulation with rituximab, nine weekly intravenous methylprednisolone infusions, methotrexate, and hydroxychloroquine. Over a ten-week period, he had dramatic improvement in his psychiatric symptoms (CY-BOCS fell from 32 to 8; CGAS improved from 15 to 80), and his suicidality abated. His eating returned to normal patterns, and his central sleep apnea resolved based on the follow-up PSG. He eventually resumed full-time private school with excellent grades and participation in active sports (basketball and soccer). While midodrine had been prescribed for POTS symptoms, the patient only took a few doses because it made him tired. During routine follow-up visits (every 1–3 months for 3 years) he denied noticeable POTS symptoms, no longer endorsing lightheadedness on prolonged standing. However, while on the immunomodulation, he had a mild relapse of OCD and POTS symptoms (lightheadedness and itchy legs) which prompted escalation of immunomodulation (addition of IVIG 60 grams monthly x 6 months) which was followed by complete resolution of OCD and POTS symptoms.

## Results

**Figure 1:**
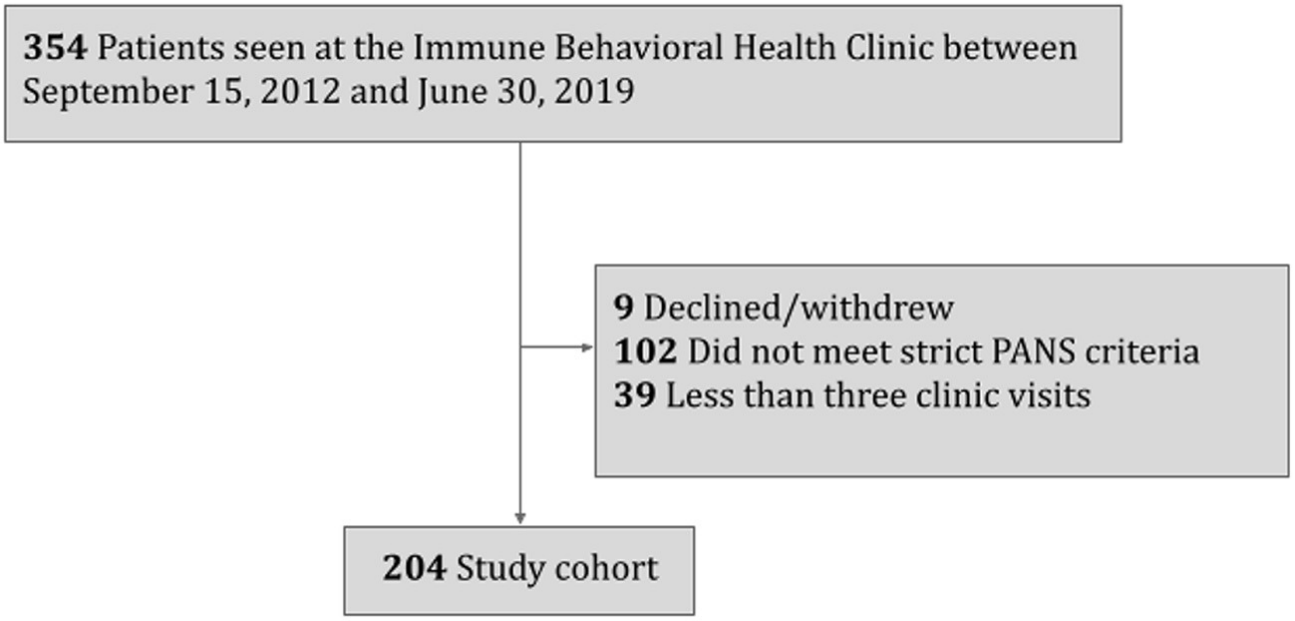
Inclusion and exclusion flowchart for a study investigating postural orthostatic tachycardia syndrome (POTS) in patients with pediatric acute-onset neuropsychiatric syndrome (PANS).

The entire study cohort included 204 eligible study subjects (Figure 1). The mean age of PANS onset was 8.6 years (SD 3.6 years) (Table 1). On average, patients presented to our IBH/PANS clinic 2 years after initial PANS symptom onset. The study cohort was predominantly male (123/204, 60%) and non-Hispanic White (158/204, 78%). Obsessive compulsive symptoms, anxiety, emotional lability, depression and aggression were the most common presenting symptoms.

**Table 1:** Characteristics of 204 consecutive patients with pediatric acute-onset neuropsychiatric syndrome (PANS) included in this study of postural orthostatic tachycardia syndrome (POTS).

	**Patients presented with PANS (*n* = 204)**
Age (years) at PANS onset, mean ± SD	8.6 ± 3.6
Age (years) at the first clinic visit, mean ± SD	10.7 ± 4.2
Follow-up time (years), mean ± SD	2.9 ± 1.8
Male gender, N (%)	123 (60%)
Non-Hispanic White,^a^ N (%)	158 (78%)
Psychiatric symptoms at the initial clinic presentation	
Obsessive compulsive symptoms	163 (80%)
Eating restriction	88 (43%)
Anxiety	168 (82%)
Emotional lability and/or depression	124 (61%)
Irritability, aggression and/or severely oppositional behaviors	125 (61%)
Behavioral/developmental regression	70 (34%)
Cognitive impairment	96 (47%)
Sensory issues	87 (43%)
Motor issues	103 (50%)
Urinary symptoms	106 (52%)
Sleep disturbances	47 (23%)
Orthostatic vitals checked clinic visit,^b^ N (%)	103 (51%)
Documented POTS anytime,^c^ N (%)	19 (9%)
Persistent POTS,^d^ N (%)	5 (2%)

a *Races of other patients included African American (n = 1), Asian (n = 8), other (n = 22), and unknown (n = 15)*.

b *Orthostatic vitals (from lying to quiet standing in 10 minutes) were checked when patients reported POTS symptoms or a change in disease status*.

c *POTS was defined by the presence of orthostatic intolerance symptoms like lightheadedness or palpitations when assuming the upright position, and an exaggerated heart rate increase associated with postural change from lying to standing. Heart rate increase was considered to be exaggerated in the following scenarios: (1) an increase over supine of ≥ 40/min after 5-10 minutes of quiet standing or upright tilt, or (2) a sustained heart rate increase of ≥ 130/min (for age ≤ 13 years) or ≥120/min (for age >13 years)*.

d *POTS was defined by persistent abnormal orthostatic vitals at least six months apart, along with persistent POTS symptoms or continuation of medications for their POTS*.

Races of other patients included African American (n=1), Asian (n=8), other (n=22), and unknown (n=15).

**Figure 2:**
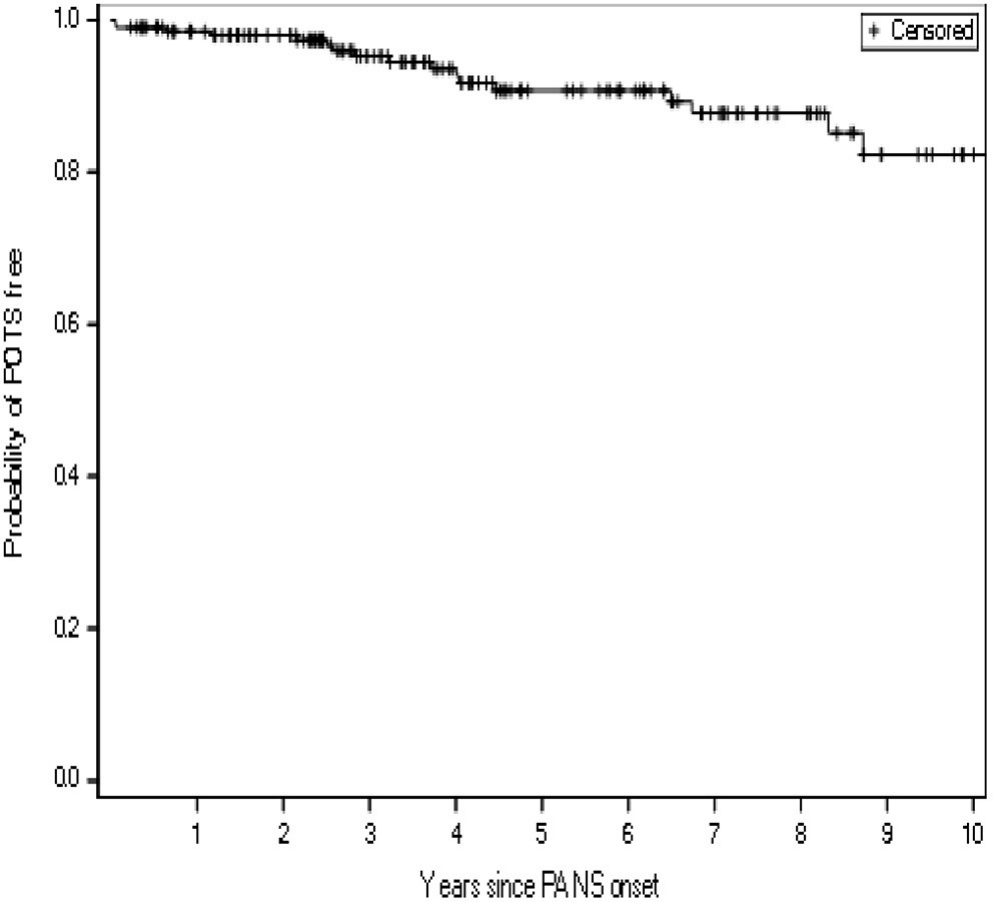
Kaplan-Meier curve showing the time-dependent risk of developing postural orthostatic tachycardia syndrome (POTS), for a study cohort of 204 consecutive patients with pediatric acute-onset neuropsychiatric syndrome (PANS)^a^. ^a^Patients were censored at their last follow-up visit.

Orthostatic vitals (blood pressure and pulse measurement on postural change from lying to 10-min standing) were documented when POTS symptoms or a change in disease states were noted (103/204, 51%) (Table 1). Evidence of POTS on orthostatic vitals was observed in 9% of our cohort. Among these patients, five (5/19, 26%) had persistent POTS, which we defined as abnormal orthostatic vitals measured at least 6 months later along with persistent POTS symptoms or continuation of medications for their POTS. The risk of POTS over time since the onset of PANS was gradual (Figure 2).

During the first few years of our clinic operation, we were not routinely screening for POTS (since patients with PANS have a variety of unexplained somatic symptoms). Therefore, we did a comparison between patients who had orthostatic vitals documented and those who did not. This comparison showed that the age of PANS onset, age of first clinic visit, duration of follow-up, demographic data, and family history did not differ (Supplementary Table 1). However, patients who had documented orthostatic vitals had higher BMI at initial clinic presentation (19.6 vs 17.0, *p* = 0.05), and their psychometric test scores (CY-BOCS, MOAS, CGBI, and CGAS) revealed higher psychiatric symptom impairment.

Table 2 shows a comparison of demographic and clinical characteristics between patients with and without evidence of POTS on orthostatic vitals. A higher proportion of males was observed in patients with POTS than in patients without POTS (79 vs 55%, *p* = 0.05). Patients with POTS also had more comorbid joint hypermobility (63 vs 37%, *p* = 0.04) and chronic fatigue (42 vs 18%, *p* = 0.03). A family history of chronic fatigue, POTS, and palpitations/syncope was also more common in patients with POTS than in patients without POTS. We did not find hypovolemia to be a potential cause of orthostatic vitals abnormality; our patients had normal serum sodium, normal serum potassium, and normal urine specific gravity regardless of orthostatic vital signs. BMI at initial clinic presentation and weight loss in the preceding three months of the orthostatic vitals were comparable between groups.

**Table 2:** Comparison of demographic and clinical characteristics between patients who had and did not have evidence of postural orthostatic tachycardia syndrome (POTS) on orthostatic vital signs.

	**Evidence of POTS (*N* = 19)**	**No evidence of POTS (*N* = 84)**	***P* value^**a**^**
Age (years) at PANS onset, mean ± SD	9.9 ± 3.2	8.5 ± 3.4	0.10
Age (years) at the first clinic visit, mean ± SD	12.4 ± 4.1	10.3 ± 4.1	0.07
Follow-up time (years), mean ± SD	3.4 ± 1.7	3.0 ± 1.8	0.31
Male gender, N (%)	15 (79%)	46 (55%)	0.05
Non-Hispanic White, N (%)	15 (79%)	69 (82%)	0.75
BMI (kg/m^2^) at the initial clinic presentation, median ± IQR	19.0 ± 9.1	19.3 ± 4.4	0.98
Weight loss in the three months preceding first clinic visit^b^	13 (68%)	43 (51%)	0.17
**Comorbidities** ^**c**^			
Joint hypermobility	12 (63%)	31 (37%)	0.04
Headache	11 (58%)	39 (46%)	0.28
Gastrointestinal symptoms like nausea, vomiting, non-specific abdominal pain, etc	10 (53%)	46 (55%)	0.99
Depression	10 (53%)	40 (48%)	0.57
Anxiety	7 (37%)	57 (68%)	0.02
Sleep problems	6 (32%)	43 (51%)	0.16
Chronic fatigue	8 (42%)	15 (18%)	0.03
Cognitive impairment	11 (58%)	44 (52%)	0.53
**Family history, N (%)**			
Chronic fatigue	1 (5%)	3 (4%)	0.56
POTS	3 (6%)	3 (4%)	0.07
Palpitations or syncope	2 (11%)	0	0.03

a *P-values were calculated for categorical variables using Chi-square tests or Fisher's exact test when appropriate. P-values were calculated for continuous variables using two-sample T-tests for normally distributed data and Wilcoxon rank-sum tests for skewed data*.

b *Weight loss in the three months prior to the date of abnormal orthostatic vitals indicative of POTS or prior to the first orthostatic vitals in patients without POTS*.

c *Comorbidities were measured at the time of abnormal orthostatic vitals indicative of POTS or at the time of the first orthostatic vital test in patients without POTS*.

Among the 19 patients with POTS, global functioning was poor (65.0 ± 40.0 vs. 52.0 ± 21.0) and caregiver burden was high (34.0 ± 17.5 vs. 39.0 ± 19.5) at the time of abnormal orthostatic vitals when compared to measurements at clinic presentation (Table 3).

**Table 3:** Comparison of psychometric test scores at the initial clinic presentation and at the time of POTS diagnosis in 19 patients with postural orthostatic tachycardia syndrome (POTS).

**Psychometric test**	**At initial clinic presentation,** **median ±IQR**	**At the time of abnormal orthostatic vitals indicative of POTS, median ±IQR**
CY-BOCS^a^	11.0 ± 21.0	11.0 ± 19.0
MOAS^b^	1.0 ± 4.0	1.0 ± 4.0
CGBI^c^	34.0 ± 17.5	39.0 ± 19.5
CGAS^d^	65.0 ± 40.0	52.0 ± 21.0

a *CY-BOCS (Children's Yale Brown Obsessive Symptom Checklist) is a measure of obsessive compulsive symptom severity (34). It ranges from 0–40; the higher the worse*.

b *MOAS (Modified Overt Aggression Scale) is a measure of aggression and opposition (35). It ranges from 0–100; the higher the worse*.

c *CGBI (Caregiver Burden Inventory) is a measure of caregiver burden. It ranges from 0–96; the higher the worse (37). A score of 36 or higher indicates the need for respite care*.

d *CGAS (Children's Global Assessment Score) is a clinician-rated measure of global functioning of the child in the past week (38). It ranges from 1–100; the higher the better*.

Unadjusted logistic regression model showed that the patient being in a PANS flare was associated with abnormal orthostatic vitals (odds ratio [OR] 3.3, 95% confidence interval [CI] 1.4–7.6, *p* < 0.01). The odds ratio was similar after adjusting for gender and BMI at clinic presentation.

Fourteen of 19 patients with POTS and PANS had resolution of POTS during the study period. The time of POTS resolution ranged from 1 to 24 months, with a median time of 1.5 months (interquartile range, 2.5 months). Seven patients improved after immunomodulation, which was employed to treat PANS, including high dose IVIG, methylprednisolone, high dose oral prednisone, rituximab, and methotrexate; these patients did not receive any specific drugs for POTS. Two patients received POTS-specific treatment, including fludrocortisone and midodrine, without immunomodulation. The five remaining patients had spontaneous resolution of POTS. Five patients had persistent POTS despite treatments with fludrocortisone, midodrine, immunomodulation, or a combination of all these.

## Discussion

The case described represents a patient with PANS and its typical features: non-Hispanic White male with pre-pubertal PANS onset; neuropsychiatric deterioration; relapsing/remitting symptoms; and comorbid autoimmune diseases. He experienced lightheadedness on prolonged standing and endorsed brain fog, anxiety, and fatigue, which have been described as major presenting symptoms of POTS (26, 27, 40). The POTS symptoms, abnormal orthostatic vitals, psychiatric symptoms, and central sleep apnea all resolved coincidentally with immunomodulatory therapies without the intervention of POTS specific therapies or changes in psychiatric medications.

Our study is the first to report the prevalence of POTS in patients with PANS and describe characteristics of patients with PANS with and without POTS. In our sample, the prevalence of POTS (9%) was higher than in the general population (1%) (31, 41). Additionally, contrary to a female predominance in POTS in the general population, males with POTS predominated in our PANS cohort. Our study also shows that a higher proportion of PANS patients with POTS had comorbid joint hypermobility, chronic fatigue, and a family history of chronic fatigue, POTS, and palpitations or syncope. These findings of comorbidity and family history are consistent with other studies (42–46), and we believe that this data would be helpful to clinicians in counseling families.

Opportunities to diagnose POTS in our study cohort fell within patients' appointments in our IBH/PANS clinic (Figure 2), at which times patients reported current symptoms, such as severe emotional and behavioral disturbances, and the multidisciplinary team including pediatricians, rheumatologists, immunologists, psychiatrists, psychologists, and nurse practitioners extensively explored their medical and psychiatric symptoms. Other symptoms may have moved to the forefront of patients' noted complaints, and clinicians' scrutiny of multiple complaints, or another mechanism, might explain why POTS was only detected years after the onset.

POTS may result from multiple mechanisms, and therefore can be broken into different subtypes, including hyperadrenergic, hypovolemic, neuropathic, mast cell-activated, and autoimmunogenic, with the first three subtypes accounting for most of the POTS cases in the general population (47). In our study, hypovolemia seems unlikely; no significant differences in serum sodium levels, serum potassium levels, or body mass index were noted between patients with and without POTS. Weight loss in the 3 months preceding orthostatic vitals was common and occurred at similar rates in PANS patients with and without POTS. We did not conduct additional laboratory tests to explore the hyperadrenergic and neuropathic signs in our patients because we did not suspect these to be contributing factors, but this might be of interest in future studies. Among our 19 patients with POTS, 10 patients had dermatographia and three patients had urticaria at the time of POTS diagnosis. It is speculated that mast cell activation may occur in some of our patients, and previous studies have shown that some psychiatric symptoms in patients with PANS improved with antihistamines (48, 49).

Evidence suggests that physical deconditioning is an important aspect of POTS syndrome and can worsen symptoms (50). In the case that we presented and in our retrospective cohort, both the symptoms from PANS and POTS often lead to inactivity and deconditioning, and this deconditioning may further contribute to POTS. Some patients who develop PANS and/or OCD continue participating in sports and physical activity despite having psychiatric symptoms, but in others, the symptoms from PANS and/or POTS are so disabling that patients stop engaging in all physical activity and become profoundly deconditioned. Most patients had their first clinic visit at least 3 months after onset, so when we identified POTS, we could not ascertain whether the POTS condition was more so a consequence of an autoimmune condition or deconditioning from chronic inactivity. However, POTS is a common sequelae for which deconditioning is just one of the number of overlapping pathologies that may precipitate orthostatic intolerance (47, 51).

In our cohort, post-infectious inflammation/autoimmunity is a likely connection between POTS and PANS. For PANS, a growing body of evidence has linked the condition and its cardinal symptom of OCD to autoimmunity and immune dysregulation (3, 10, 52–58). In patients with POTS, evidence of immune dysregulation points to dysfunction of the cholinergic anti-inflammatory pathway, whereby a reduction in signaling of the alpha-7 subunit of nicotinic acetylcholine receptors (which are expressed in T cells, B cells, monocytes and endothelial cells) was associated with a subsequent reduction in the release of anti-inflammatory cytokines (59–63). More recently, patients with the coronavirus disease 2019 (COVID-19) have been reported to develop psychiatric symptoms (64, 65) and autonomic dysfunction, (66). Of note, the first case of POTS developing several months after confirmed SARS-CoV-2 infection was reported in September 2020 (67), and the first case of PANS developing after SARS-CoV-2 was reported in May 2021 (68). With infection being a well-described trigger of POTS and a suspected trigger of PANS, the magnitude of the COVID-19 pandemic represents a unique opportunity to understand the pathophysiological mechanisms of post-infectious neurological and neuropsychiatric sequelae.

Our preliminary analysis shows that in a PANS flare, patients had three-fold increased odds of having orthostatic vital sign abnormality with POTS symptoms, a finding that should be explored in larger populations to allow the control of confounders. In other autoimmune conditions, such as multiple sclerosis, disease severity and disease progression have also been found to predict the presence of autonomic dysregulation (69). If the association between inflammation and dysautonomia holds true, it may also explain why POTS resolved after the start of immunomodulatory therapies in the majority of our patients with POTS and PANS.

Our study has limitations. First, we were unable to routinely check orthostatic vitals for every patient at every clinic visit, possibly resulting in selection bias. Patients with and without documented orthostatic vitals were comparable in many aspects; however, those without documented orthostatic vitals had higher rates of anxiety and worse psychometric test scores. This illustrates a practical challenge in our clinic as patients who are doing poorly from the mental health standpoint may not be able to cooperate with orthostatic vital measurement. Patients with comorbid anxiety at a visit were statistically less likely to have had orthostatic vital signs measured (*p* = 0.01, Supplementary Table 1).

Secondly, POTS symptoms, such as subjective fatigue, brain fog, cognitive impairment, concentration problems, anxiety, palpitations, overlap with symptoms often reported by patients affected with PANS. As a result, POTS symptoms may be overlooked, and its prevalence may have been underestimated in our sample. To accurately identify POTS, we could assess the autonomic symptoms in a more comprehensive and systematic way, with an instrument such as the Composite Autonomic Symptom Score (COMPASS-31), a questionnaire assessing symptoms of autonomic dysfunction (70).

## Conclusion

This study describes a higher prevalence of POTS in patients with PANS than in the general pediatric population and an association of POTS with comorbidities such as joint hypermobility, anxiety, chronic fatigue, and family history of cardiovascular issues. We also observed that POTS was more likely observed during a PANS flare. It is interesting that immune dysfunction, which is present in PANS, may be implicated in some individuals with PANS and POTS. Future studies investigating these patients will shed light on their coincidence, pathophysiology and prognosis.

## Data Availability Statement

The raw data supporting the conclusions of this article will be made available by the authors, without undue reservation.

## Ethics Statement

The studies involving human participants were reviewed and approved by Stanford Institutional Review Board. Written informed consent to participate in this study was provided by the participants' legal guardian/next of kin. Written informed consent was obtained from the minor(s)' legal guardian/next of kin for the publication of any potentially identifiable images or data included in this article.

## Author Contributions

AC and JF contributed to conception and design of the study. AC, JG, and MH organized the database. AC performed the statistical analysis. AC, JF, and MH wrote the first draft of the manuscript. SJ and JG wrote sections of the manuscript. All authors contributed to manuscript review, read, and approved the submitted version.

## Funding

Supported in part by the Stanford University Human Biology Research Exploration Program (HB-REX). This sponsor did not partake in this study.

## Conflict of Interest

The authors declare that the research was conducted in the absence of any commercial or financial relationships that could be construed as a potential conflict of interest.

## Publisher's Note

All claims expressed in this article are solely those of the authors and do not necessarily represent those of their affiliated organizations, or those of the publisher, the editors and the reviewers. Any product that may be evaluated in this article, or claim that may be made by its manufacturer, is not guaranteed or endorsed by the publisher.
