# Supplementary material for: Children With PANS May Manifest POTS
Source: Front Neurol. 2022 Apr 26;13:819636. doi: 10.3389/fneur.2022.819636 (PMC9086964; doi:10.3389/fneur.2022.819636)
Supplement: Supplementary file 1 [file Table_1.pdf]

Appendix Table 1. Comparison between patients who had and did not have orthostatic vitals documented in the charts.

	Orthostatic vitals documented (n=103)	Orthostatic vitals never documented (n=101)	P-value[a]
Age (yrs) at PANS onset, mean ± SD	8.8 ± 3.4	8.4 ± 3.7	0.38
Age (yrs) at first clinic visit, mean ± SD	10.7 ± 4.1	10.7 ± 4.2	0.93
Follow-up (years), mean ± SD	3.1 ± 1.8	2.7 ± 1.8	0.14
Male sex, N (%)	61 (59%)	62 (61%)	0.75
Non-Hispanic White, N (%)	84 (82%)	74 (73%)	0.16
BMI (kg/m^2) at initial clinic presentation, median ± IQR	19.6 ± 6.2	17.0 ± 4.1	0.05
Comorbidities[b]			
Joint hypermobility	43 (42%)	32 (32%)	0.14
Headache	50 (49%)	49 (49%)	0.94
Gastrointestinal symptoms (nausea, vomiting, abdominal pain, etc)	56 (54%)	59 (58%)	0.61
Depression	50 (49%)	48 (48%)	0.83
Anxiety	64 (62%)	80 (79%)	0.01
Sleep problems	49 (48%)	41 (41%)	0.28
Chronic fatigue	23 (22%)	13 (13%)	0.08
Cognitive impairment	55 (53%)	64 (63%)	0.17
Family history, N (%)			
Chronic fatigue	4 (4%)	7 (7%)	0.34
POTS	6 (6%)	5 (5%)	0.78
Palpitations/syncope	2 (2%)	1 (1%)	1.00
Psychometric scale scores at initial clinic presentation, median ± IQR			
CY-BOCS[c]	15.0 ± 15.0	14.0 ± 9.0	0.22
MOAS[d]	1.0 ± 9.0	0 ± 3.0	0.07
CGBI[e]	32.0 ± 31.0	27.0 ± 29.0	0.19
CGAS[f]	53.0 ± 34.0	67.0 ± 30.0	0.27

[a]P-values were calculated by Chi-square tests or when appropriate, Fisher's exact test for categorical variables. For continuous variables, two-sample T-test for normally distributed data and Wilcoxon rank-sum test for skewed data were conducted.
[b]Comorbidities were measured at the time of the first orthostatic vital test in tested patients, and at the time of clinic presentation in non-tested patients.
[c]CY-BOCS (Children's Yale Brown Obsessive Symptom Checklist) is a measure of obsessive compulsive symptom severity.(Scahill et al. 1997) It ranges from 0-40; the higher the worse. **Since PANS is a relapsing-remitting illness, many patients come to their first appointment in a resolved relapse, while others come during a relapse, which explains the wide CYBOCS range.**
[d]MOAS (Modified Overt Aggression Scale) is a measure of aggression and opposition.(Giles and Mohr 2007) It ranges from 0-100; the higher the worse.

[e]CGBI (Caregiver Burden Inventory) is a measure of caregiver burden. It ranges from 0-96; the higher the worse.(Frankovich et al. 2018) A cut-off point of 36 indicates respite care.
[f]CGAS (Children's Global Assessment Score) is a clinician-rated measure of global functioning of the child in the past week.(Shaffer et al. 1983) It ranges from 1-100 with higher scores reflecting more superior functioning/health.
